# Development and Validation of Three Regional Microsimulation Models for Predicting Colorectal Cancer Screening Benefits in Europe

**DOI:** 10.1177/2381468320984974

**Published:** 2021-01-29

**Authors:** Andrea Gini, Maaike Buskermolen, Carlo Senore, Ahti Anttila, Dominika Novak Mlakar, Piret Veerus, Marcell Csanádi, Erik E. L. Jansen, Nadine Zielonke, Sirpa Heinävaara, György Széles, Nereo Segnan, Harry J. de Koning, Iris Lansdorp-Vogelaar

**Affiliations:** Department of Public Health, Erasmus MC, University Medical Center Rotterdam, Rotterdam, The Netherlands; Department of Public Health, Erasmus MC, University Medical Center Rotterdam, Rotterdam, The Netherlands; SC Epidemiology, Screening, Cancer Registry, Città della Salute e della Scienza University Hospital, CPO, Turin, Italy; Finnish Cancer Registry, Helsinki, Finland; National Institute for Public Health, Ljubljana, Slovenia; National Institute for Health Development, Tallinn, Estonia; Syreon Research Institute, Budapest, Hungary; Department of Public Health, Erasmus MC, University Medical Center Rotterdam, Rotterdam, The Netherlands; Department of Public Health, Erasmus MC, University Medical Center Rotterdam, Rotterdam, The Netherlands; Finnish Cancer Registry, Helsinki, Finland; Syreon Research Institute, Budapest, Hungary; SC Epidemiology, Screening, Cancer Registry, Città della Salute e della Scienza University Hospital, CPO, Turin, Italy; Department of Public Health, Erasmus MC, University Medical Center Rotterdam, Rotterdam, The Netherlands; Department of Public Health, Erasmus MC, University Medical Center Rotterdam, Rotterdam, The Netherlands

**Keywords:** colorectal cancer screening, microsimulation modeling, model calibration, model validation

## Abstract

**Background.** Validated microsimulation models have been shown to be useful tools in providing support for colorectal cancer (CRC) screening decisions. Aiming to assist European countries in reducing CRC mortality, we developed and validated three regional models for evaluating CRC screening in Europe. **Methods.** Microsimulation Screening Analysis–Colon (MISCAN-Colon) model versions for Italy, Slovenia, and Finland were quantified using data from different national institutions. These models were validated against the best available evidence for the effectiveness of screening from their region (when available): the Screening for COlon REctum (SCORE) trial and the Florentine fecal immunochemical test (FIT) screening study for Italy; the Norwegian Colorectal Cancer Prevention (NORCCAP) trial and the guaiac fecal occult blood test (gFOBT) Finnish population-based study for Finland. When published evidence was not available (Slovenia), the model was validated using cancer registry data. **Results.** Our three models reproduced age-specific CRC incidence rates and stage distributions in the prescreening period. Moreover, the Italian and Finnish models replicated CRC mortality reductions (reasonably) well against the best available evidence. CRC mortality reductions were predicted slightly larger than those observed (except for the Florentine FIT study), but consistently within the corresponding 95% confidence intervals. **Conclusions.** Our findings corroborate the MISCAN-Colon reliability in supporting decision making on CRC screening. Furthermore, our study provides the model structure for an additional tool (EU-TOPIA CRC evaluation tool: http://miscan.eu-topia.org) that aims to help policymakers and researchers monitoring or improving CRC screening in Europe.

Microsimulation models have been shown to be useful tools in quantifying benefits and harms of colorectal cancer (CRC) screening,^1–4^ but decision makers should be prudent in determining how much confidence to place in the results of those models.^5,6^ Several microsimulation models are currently used to inform CRC screening programs,^6^ but few have been extensively validated.^7,8^ The Microsimulation Screening Analysis–Colon (MISCAN-Colon) model is among this latter group, with extensive published information on its model structure, assumptions, and validation.^9,10^

The MISCAN-Colon simulates the sequence that leads from adenoma to clinical CRC detection, incorporating parameters based on published data, such as adenoma prevalence and lifetime CRC incidence.^11–14^ Other crucial parameters, such as duration times from adenoma formation to CRC, are impossible to observe—in an ethically acceptable manner—and could not be directly based on existing evidence. Those parameters were, however, inferred using results from randomized control trials (RTCs) investigating the effectiveness of CRC screening.^8,15–17^

Although the MISCAN-Colon model structure has been validated,^16^ it is still unclear how reliable the MISCAN-Colon results may be considering different populations. CRC incidence rates varied remarkably across countries,^14^ and this may be caused by variations in the underlying cancer risk (due to genetics, lifestyle, and socioeconomic factors possibly affecting natural history of the disease). Thus, the EU-TOPIA project (EU-Framework Programme, Horizon 2020–634753) decided to investigate the robustness of the MISCAN-Colon structure across a variety of countries, aiming to further generalize and use the MISCAN-Colon model across Europe. The final objective of EU-TOPIA is to develop a standardized online version of the model (EU-TOPIA CRC evaluation tool; http://miscan.eu-topia.org) that allows European policymakers to quantify and predict CRC screening benefits in their country.

In this study, we developed and validated three European regional model versions, providing modelling results that support the reliability of MISCAN-Colon and the robustness of its assumptions and model structure.

## Materials and Methods

### MISCAN-Colon

MISCAN-Colon is a stochastic microsimulation model that simulates the life histories of many individuals from birth to death. In each simulated individual, zero, one, or more than one adenoma may occur. These adenomas might progress in size and might become malignant. Survival after CRC diagnosis is modelled depending on age, stage, and localization of cancer at diagnosis. Screening may alter the simulated life histories, detecting some CRCs at an earlier stage or preventing them by removing a precancerous lesion. Comparing life histories with and without screening, MISCAN-Colon quantifies the effectiveness of CRC screening. In Europe, it has been used to design, monitor, and evaluate the Dutch CRC screening program, predicting its future benefits.^4^ The Dutch MISCAN-Colon model version was calibrated to age- and stage-specific (UICC TNM stage classification) CRC incidence rates observed in the Netherlands in 1999-2003 (see Supplementary Methods for more information on the MISCAN-Colon structure, underlying assumptions, and results of the Dutch model version).^18^ Survival rates were based on data from the South of the Netherlands.^19^ Specific model parameters, such as adenoma dwell time and the preclinical duration of CRC, were calibrated replicating outcomes of CRC screening RCTs^8^ and, subsequently, validated to the results of the NORCCAP trial.^16^

### Development of Country-Specific MISCAN-Colon Model Versions for Italy, Slovenia, and Finland

In this study, we developed three new country-specific model versions (Italy, Slovenia, and Finland) that along with the Dutch model version are aiming to be representative of all regions in Europe (Italy for the Southern; the Netherlands for Western; Slovenia for Eastern; and Finland for Northern European countries). Italy, Slovenia, and Finland were countries with research institutions directly involved in the EU-TOPIA project. In developing each model, we used a specific calibration process (Supplementary Methods). Briefly, we started using the previously calibrated Dutch model version, adjusting demographic and CRC epidemiological assumptions such as population size, all-cause mortality, CRC relative survival by stage, and cancer localization (using the country-specific data in the period before the introduction of screening). Then, we initially calibrated the model parameters behind age-specific adenoma onset and distribution of CRC stages (parameters that lead to country-specific CRC incidence and stage distribution), assuming the same parameters of the Dutch model for the adenoma progression and the preclinical duration of CRC. Calibration was performed using country-specific CRC incidence rates and CRC stage distribution (reported in the prescreening period). We first validated the model versions replicating the data that were used in the calibration process (internal validation). Then, the models were externally validated (i.e., model replication of data not used in calibrating the models) against the best evidence for screening effectiveness for the respective countries (when the evidence was available, i.e., for Italy and Finland). A decision algorithm was used to select the evidence to perform an external model validation: four published studies were selected from a systematic review assessing the effectiveness of screening on CRC mortality in Europe (see Supplementary Methods for more details on the selection of the studies). In order to perform an external validation, the models were accordingly adjusted to replicate the study-specific population and screening pattern. When evidence for screening effectiveness was not available in the specific country or its European region (i.e., Slovenia), model versions were only internally validated. If models failed internal or external validation, we reiterated the developing process relaxing the assumption on adenoma and CRC progression parameters (by re-calibrating these parameters as well).

Data used for calibration, internal validation, and external validation are reported below for each model version and, extensively, in Table 1 and Table 2. Primary validation targets were CRC incidence and mortality rates and stage distribution observed in the prescreening period for internal validation; and CRC mortality reductions due to screening for the external validation (CRC incidence reductions were also investigated and reported in Supplementary Methods). In the (internal or external) validation procedures, a model replication was considered “consistent” when the simulated model prediction was estimated within the 95% confidence intervals (95% CI) of the corresponding observed outcome. When 95% CIs were not reported in the study or in the data source, those were computed assuming Poisson or binomial distributions.

**Table 1:** Key Calibration Modelling Assumptions

Assumptions for Calibration/Internal Validation	MISCAN-Colon Model		
	Italy	Slovenia	Finland
Demography			
All-cause mortality	Italian Life Tables, 1998 Source: Human Mortality Database^24^	Slovenian Life Tables, 2008 Source: Human Mortality Database^24^	Finnish Life Tables, 1999 Source: Human Mortality Database^24^
Population	Italian Population in 1998 Source: Human Mortality Database^24^	Slovenian Population in 2008 Source: Human Mortality Database^24^	Finnish Population in 1999 Source: Human Mortality Database^24^
Natural history of CRC			
Adenoma onset (*calibrated*)	Age-dependent (nonhomogeneous Poisson)^a^	Age-dependent (nonhomogeneous Poisson)^a^	Age-dependent (nonhomogeneous Poisson)^a^
Adenoma progression			
State transitions		Age-dependent (source: Rutter et al.^8^)^b^		
State durations, years (total)		Exp(1/λ = 140) (source: Rutter et al.^8^)^b^		
Cancer progression (preclinical)			
Stage transitions		Age-dependent (source: Rutter et al.^8^)^b^		
Stage durations, years		Exp(1/λ = 2.5) (source: Rutter et al.^8^)^b^		
Colorectal cancer incidence (without exposure to screening, *calibrated*)	Age-/stage-dependent^a^ Period: 1998–2002 Source: IARC CI5-IX^46^	Age-/stage-dependent^a^ Period: 2004–2008 Source: Slovenian CR^13^	Age-/stage-dependent^a^ Period: 1999–2003 Source: Finnish CR^12^
Colorectal cancer stage distribution (without exposure to screening, *calibrated*)	Age-dependent Period: 2000–2008 (no screening) Source: IMPATTO COLONRETTO^47^	Age-dependent Period: 2004–2008 Source: Slovenian CR^13^	Age-dependent Period: 1999–2003 Source: Finnish CR^12,c^
Colorectal cancer survival	Age-/stage-dependent Period: 1997–1999 Source: EUROCARE^25,e^	Age-/stage-dependent Period: 2000–2007 Source: EUROCARE^27,e^	Age-/stage-dependent Period: 2000–2007 Source: EUROCARE^27,e^
Colorectal cancer localization	Period: 1998–2002 Source: IARC CI5-IX^46^	Period: 2004–2008 Source: Slovenian CR^13^	Period: 1999–2003 Source: Finnish CR^12^
Colorectal cancer mortality^d^	Age-dependent Period: 1998–2002 Source: ISS-ISTAT^48^	Age-dependent Period: 2004–2008 Source: Slovenian CR^13^	Age-dependent Period: 1999–2003 Source: Finnish CR^12^

**Figure 1:**
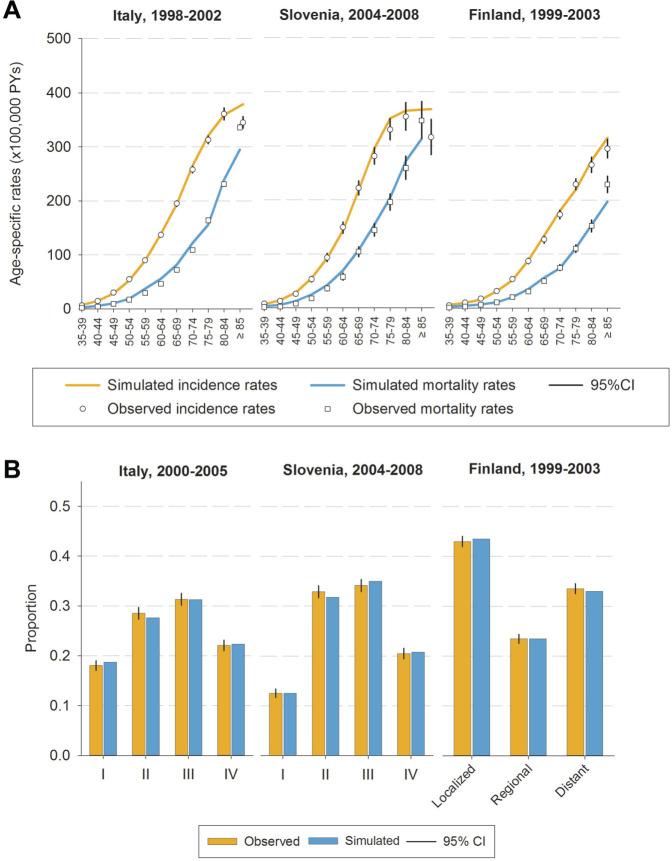
Simulated and observed colorectal cancer incidence, mortality rates (A), and stage distributions (B) in the period before the introduction of screening in Italy (1998–2002), Slovenia (2004–2008), and Finland (1999–2003). CRC, colorectal cancer.

Calibrated, parameters calibrated with country-specific data; CR, cancer registry; CRC, colorectal cancer; Exp, exponential distribution.

a Calibrated together with colorectal cancer incidence in the prescreening period (please see Supplementary Tables 2 for more detailed information).

b Parameters assumed equals to those previously calibrated in Rutter et al. and validated in Buskermolen et al.^8,16^

c Stage distribution was adjusted considering a different staging system (No UICC TNM but Localized, Regional, Distant categorization).

d Data on colorectal cancer mortality was not used inside the model (only for graphical inspection in Figure 1).

e Survival rates after CRC diagnosis were adjusted based on source data (adjustment details in Supplementary Methods).

**Table 2:** Key Validation Modelling Assumptions^a^

Validation Assumptions	Italy	Finland
Selected Study (Endoscopy Screening)	SCORE Trial^21^	NORCCAP Trial^29^
Demography		
All-cause mortality	Italian Life tables, 1995 (30% lower all-cause mortality, individuals were willing to participate in the study—assumed healthier than the general population) Source: Demo-ISTAT^49^	Norwegian life tables, 2005 Source: Statistics Norway
Population	Age: 55–64	Age: 50–65
Randomization	Individuals randomized in 1997 (middle year in the study: 1995–1999)	Birth cohorts from 1935 to 1945 screened in 1999–2000; and birth cohorts from 1946 to 1950 in 2001
CRC natural history in the study population		
Adenoma onset + CRC incidence rates + CRC stage distributions + CRC relative survival	Italian model parameters	Finnish model parameters. Adenoma onset/CRC incidence assumed 57% more elevated (multiplicative factor) to match the differences in CRC incidence between Norway and Finland in the prescreening period. CRC stage distribution and CRC relative survival were adjusted according to Norwegian CR data (as assumed in the model validation performed by Buskermolen et al.^16^)
Screening cohort		
Definition	Eligible individuals, invited to screening	Individuals invited to screening. Compared to control group, CRC risk was assumed 3% lower in those that attended screening and 5% higher in those who did not attend (as assumed in the model validation performed by Buskermolen et al.^16^)
Screening test	Flexible-sigmoidoscopy	Flexible-sigmoidoscopy or flexible-sigmoidoscopy in combination with a single FIT
Screening interval	Once in a lifetime at randomization. However, 65% screened in the year of randomization; 28% after 1 year; and 7% after at least 2 years following data sent by the trial’s authors (personal communication)	Once in a lifetime at randomization
Adherence in screening	58.3% (Source: SCORE trial^21^)	63% (Source: NORCCAP trial^29^)
Screening sensitivity	Flexible-sigmoidoscopy (only for left colon + rectum): 75% Adenomas (≤5 mm); 85% adenomas (>5 mm); 95% cancers and large adenomas (≥10 mm) Diagnostic colonoscopy: 75% adenomas (≤5 mm); 85% adenomas (>5 mm); 95% cancers and large adenomas (≥10 mm) Source: US colonoscopy studies.^50,51^ 91% of colonoscopies reached cecum (Source: Italian FS screening^23^)	FIT: Up to 71% for cancers. Source: NORCCAP MISCAN-Colon model validation^16^ Flexible-sigmoidoscopy (only for left colon + rectum): 75% adenomas (≤5 mm); 85% adenomas (>5 mm); 95% cancers and large adenomas (≥10 mm) Diagnostic colonoscopy: 75% adenomas (≤5 mm); 85% adenomas (>5 mm); 95% cancers and large adenomas (≥10 mm) Source: US colonoscopy studies.^50,51^ 89% of colonoscopies reached cecum (Source: NORCCAP MISCAN-Colon model validation^16^)
Screening specificity	98% (Source: NORCCAP MISCAN-Colon model validation^16^)	98% (Source: NORCCAP MISCAN-Colon model validation^16^)
Adherence in diagnostic colonoscopy	93% (Source: Italian FS screening^23^)	96% (Source: NORCCAP MISCAN-Colon model validation^16^)
Diagnostic colonoscopy referral criteria	≥1 adenoma (≥6mm); and ≥3 adenomas (including <6 mm)	Any adenomas found at screening
Adherence in FU post-colonoscopy (Surveillance)	81% (Source: Italian FIT screening^23^)	80% (Source: NORCCAP MISCAN-Colon model validation^16^)
Post-colonoscopy criteria	Surveillance in: >1 adenomas (≥10 mm), 3 years; and >2 adenomas, 3 years^52^	Surveillance in: <3 adenomas (≥10 mm), 10 years; and >2 adenomas, 5 years^16^
No screening cohort (control group)		
Definition	Eligible individuals, no further contact	Individuals, not invited and not further contacted
Additional specific assumptions	—	—
**Selected Study (Stool Tests)**	**Ventura et al.^26^**	**Pitkäniemi et al.^28^**
Demography		
All-cause mortality	Florentine life tables, 1996 (Source: Demo-ISTAT^49^)	Finnish Life Tables, 2008 (Source: Human Mortality Database^24^)
Population	Age: 50–70	Age: 60–69
Randomization	Individuals randomized in 1996 (middle year in the study: 1993–1999)	Individuals randomized in 2004 (assumed as standardized starting year); screening invitation reflected the pattern designed in Malila et al.^53^)
CRC incidence in the study population		
Adenoma onset + CRC incidence rates + CRC stage distributions + CRC relative survival	Italian model parameters. Adenoma onset/CRC incidence assumed 18% more elevated (multiplicative factor) to match the differences in CRC incidence between Italy (without Tuscany) and Tuscany in the prescreening period. CRC relative survival was assumed up to 5% points higher (Source: Tuscany CR^33^ and IARC CI5-VI^46^)	Finnish model parameters. CRC relative survival adjusted with estimates reported for 2010–2012 in the Finnish CR (Source: Finnish CR^12^)
Screening cohort		
Definition	Individuals invited and screened in the first round (Source: Ventura et al.^26^)	Individuals invited to screening
Screening test	FIT (positive cutoff: 100 ng/mL)	gFOBT
Screening interval	Biennial	Biennial
Age target	50–70	60–69
Adherence in screening	100% first round, overall 40%: 3.5 average attended screening rounds (Source: Ventura et al.^26^)	69% (Source: Pitkäniemi et al.^28^)
Screening sensitivity	FIT: 0% adenomas (<6 mm); 7% adenomas (6–9 mm); 24% adenomas (≥10 mm); 63% preclinical CRCs (shortly before clinical diagnosis)^a^ CRCs; and 89% preclinical CRCs (long before clinical diagnosis)^b^ (Source: Imperiale et al.^54^ and Knudsen et al.^1^) Diagnostic colonoscopy: 75% adenomas (≤5 mm); 85% adenomas (>5 mm); 95% cancers and large adenomas (≥10 mm) Source: US colonoscopy studies^50,51^ 88% of colonoscopies reached cecum (Source: Italian FIT screening^24^)	gFOBT: <0.1% adenomas (<10 mm); 8% adenomas (≥10 mm); 56% preclinical CRCs (long before clinical diagnosis)^b^; and 24% preclinical CRCs (shortly before clinical diagnosis).^b^ Source: Parameters calibrated using data from the Finnish CRC screening program 2004–2006 (adenomas and CRC detection rates in individuals screened for the first time, Supplementary Methods) Diagnostic colonoscopy: 75% adenomas (≤5 mm); 85% adenomas (>5 mm); 95% cancers and large adenomas (≥10mm) Source: US colonoscopy studies^50,51^ 89% of colonoscopies reached cecum (Source: NORCCAP MISCAN-Colon model validation^16^)
Screening specificity	96% (Source: Imperiale et al.^5^)	98% Source: Parameter calibrated using data from the Finnish CRC screening program 2004–2006 (adenomas and CRC detection rates in individuals screened for the first time, Supplementary Methods)
Adherence in diagnostic colonoscopy	73% (Source: Ventura et al.^26^)	84% (Source: Pitkäniemi et al.^28^)
Diagnostic colonoscopy referral criteria	A positive FIT	A positive gFOBT
Adherence in FU post-colonoscopy (Surveillance)	83% (Source: Italian FIT screening,^23^ highest value reported)	84% (Assumed equal to adherence in diagnostic colonoscopy)
Post-colonoscopy criteria	Surveillance in: >1 adenomas (≥10 mm), 3 years; and >2 adenomas, 3 years. As assumed in the SCORE trial validation.^52^	Assumed surveillance in: >1 adenomas (≥10 mm), 10 years
No screening cohort (control group)		
Definition	Individuals invited and not screened in the first round (“Not Attenders”)	Individuals not invited to screening
Specific assumptions	CRC risk in “Not-Attenders” assumed 12% additionally higher than attenders (18% × 12% = 32% higher compared to Italian model parameters) “Not-attenders” were assumed to participate in screening after 1999 according to data provided in Ventura et al.^26^: 7% only to 1 screening round; 7% more than 2; 7% only 2 (average rounds attended: 0.5)	—

a Direct incorporation meant information directly incorporated in the model in Step 3 (Appendix Method 2); Fixed, no changes in the general structure of the model; Calibrated, parameters calibrated with country-specific data; CRC, colorectal cancer; CR, cancer registry; FIT, Florentine fecal immunochemical test; gFOBT, guaiac fecal occult blood test.

b In MISCAN-Colon model CRC sensitivity of stool tests are simulated considering preclinical CRC shortly and long before clinical diagnosis as described in Lansdorp-Vogelaar et al.^55^

### Data Sources for Calibration and External Validation

#### The Italian Model Version

We used the IARC cancer incidence in five continents databases (Vol. IX, period 1998–2002) to inform and calibrate the Italian model.^14^ Cancer registry data from Turin, Milan, Genoa, Florence, and Prato were excluded due to the early introduction of population-based screening programs or pilot studies in those areas (Supplementary Methods).^20–22^ Stage distribution parameters were calibrated using data from the Cancer Screening National Monitoring reports.^23^ We modelled the age distribution of the Italian population in 1998 using data from the Human Mortality Databases.^24^ CRC relative survival was adjusted as described in Supplementary Methods, using data published by EUROCARE group.^25^ Two studies were selected aiming to (externally) validate the Italian model: the “once-only” sigmoidoscopy screening or Screening for COlon REctum (SCORE) trial; and the cohort study assessing FIT screening in Florence.^21,26^ The first was a multicenter RCT conducted in Italy assessing the efficacy of FS screening (offered once in life at 55–64 years of age).^21^ Individuals were randomly assigned to intervention group (FS, *n* = 17,148) or control group (no further contact, *n* = 17,144), between 1995 and 1999. Incidence and mortality follow-up ended, respectively, December 31, 2007, and December 31, 2008. The second study was a population-based cohort study performed in Florence to test biennial FIT screening effectiveness.^26^ Subjects undergoing FIT screening between 1993 and 1999 (attenders; *n* = 6,961) were compared to unscreened individuals from the same district (invited but not attenders; *n* = 26,285). Follow-up ended on December 31, 2008.

#### The Slovenian Model Version

We calibrated the Slovenian model using CRC incidence and stage distribution data from the cancer registry of Slovenia (2004–2008, the period before implementation of organized FIT screening).^13^ The model was adjusted to simulate the Slovenian population in 2008 (based on data from the Human Mortality Databases).^24^ CRC relative survival was adjusted as described in Supplementary Methods, using data published by EUROCARE group.^27^ As no published evidence was retrieved for CRC screening effectiveness in Eastern Europe, we could not externally validate the Slovenian model.

#### The Finnish Model Version

We calibrated the Finnish MISCAN-Colon version using CRC incidence and stage distribution data observed in the Finnish Cancer Registry between 1999 and 2003,^12^ before the introduction of a population-based screening pilot study investigating the efficacy of gFOBT screening in 2004.^28^ CRC stage distribution data needed to be converted before performing the model calibration due to the different CRC staging classification in Finland (different from the UICC TNM stage classification). The conversion was performed as follows: localized CRCs were assumed for 1/3 as TNM stage I and for 2/3 as TNM stage II (based on the CRC stage proportions observed in the Netherlands, Italy, and Slovenia); regional (CRCs nonlocalized, only regional lymph node metastases or with no information on extent) as TNM stage III; and distant (CRCs metastasized further than regional lymph nodes) as TNM stage IV. We used the model to simulate the 1999 age-specific Finnish population based on data from the Human Mortality Databases.^24^ CRC relative survival was adjusted as described in Supplementary Methods, using data published by EUROCARE group.^27^ Two studies were selected to (externally) validate the Finnish model: the Norwegian Colorectal Cancer Prevention (NORCCAP) Trial and the Finnish gFOBT screening RCT.^28,29^ The first was an RCT conducted in Norway assessing the effectiveness of FS on CRC incidence and mortality (with 100,210 individuals aged 50–64 years randomized to screening or control group). Screening was performed between 1999 and 2001 and follow-up ended December 31, 2011 (10.9 years of follow-up).^29^ The second study was a large randomized study gradually performed from 2004 to 2012 in Finland. Individuals aged 60 to 69 years were randomly allocated to screening (biennial gFOBT screening; *n* = 180,210) and to a control group (*n* = 180,282).^28^ The median follow-up was 4.5 years (maximum of 8.3 years).

## Results

### Internal Validation

In the absence of screening, the model versions predicted CRC incidence and CRC stage distribution consistently in Italy, Finland, and Slovenia in the period before the introduction of screening (Figure 1). However, CRC incidence rates were slightly overestimated in Italy (1998–2002) among individuals aged 85 years or older, and in Slovenia (2004–2008) among those aged 75 to 79 or older than 85 years. CRC mortality rates (data not used for calibrating the models) were underestimated to some extent among elderly individuals in Italy (1998–2002; age groups: 75–79; and ≥85 years), in Slovenia (2004–2008; those aged 85 years or older), and in Finland (1999–2003; ≥85 years).

### External Validation

**Figure 2:**
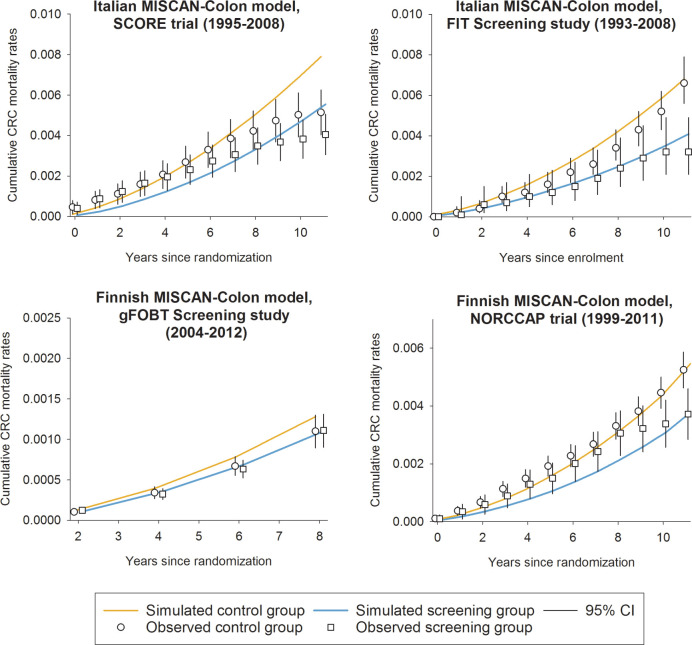
Simulated and observed cumulative colorectal cancer mortality in SCORE trial, Florentine FIT population-based screening program, NORCCAP trial, and Finnish gFOBT population-based study. CRC, colorectal cancer.

When an external validation was performed (Italy and Finland), the model versions consistently replicated CRC mortality reductions due to FS, FIT, or gFOBT screening. The Italian model version predicted a 30% lower CRC mortality after 11 years of follow-up in the intervention arm of the SCORE trial (FS), consistently with the trial outcomes (simulated relative risk [RR] = 0.70; observed RR = 0.78, 95% CI: 0.56–1.08; Table 3). Moreover, CRC mortality reduction was also consistently predicted in the Florentine FIT screening “attenders” group (36% lower compared to not attenders, RR = 0.64) after 11 years of follow-up (observed RR = 0.59, 95% CI: 0.37–0.93; Table 3). Nevertheless, CRC deaths were overestimated in replicating both screening and control arm of the SCORE trial, with CRC cumulative mortality overestimated after 9 follow-up years of the SCORE control arm and underestimated in the first 4 follow-up years of the SCORE screening arm (Figure 2). CRC cumulative incidence rates were also underestimated replicating the controls of the FIT Florentine study (after 8 years of follow-up; Supplementary Methods).

**Table 3:** Observed and Model Predicted Reductions in Colorectal Cancer Mortality Due to Screening per Model Version (Italy and Finland)

Country Model\Study\Screening Test	Outcome	Source	CRC Mortality Reduction (RR, 95%CI)	Rates per 100,000 PYs^a^	
				Control	Screened
Italian model version					
Segnan et al. (SCORE)^21^ (Sigmoidoscopy, Italy)	CRC mortality (follow-up: 11.4 years)	Estimated	0.78 (0.56–1.08)	44 (36–55)	35 (27–44)
		MISCAN-Colon (IT)	0.70	65	46
Ventura et al^26,b^ (FIT, Florence, Italy)	CRC mortality (follow-up: 10.7 years)	Estimated	0.59 (0.37–0.93)	55 (46–64)	30 (19–45)
		MISCAN-Colon (IT)	0.63	63	38
Finnish model version					
Pitkäniemi et al^28^ (gFOBT, Finland)	CRC mortality (follow-up: 4.5 years)	Estimated	1.04 (0.84–1.28)	20 (12–32)	21 (13–32)
		MISCAN-Colon (FI)	0.92	24	22
Holme et al. (NORCCAP)^29^ (sigmoidoscopy and FIT, Norway)	CRC mortality (follow-up: 10.9 years)	Estimated	0.73 (0.56–0.94)	43 (39–48)	31 (25–40)
		MISCAN-Colon (FI)	0.71	41	29

CI, confidence interval; CRC, colorectal cancer; FI, Finnish model version; FIT, immunochemical fecal test; IT, Italian model version; NORCCAP, Norwegian Colorectal Cancer Prevention Trial (Norway); PYs, person-years; RR, relative risk; SCORE, Screening for Colon Rectum Trial (Italy).

a When studies did not report CRC rates with 95% CI, those were estimated assuming a Poisson distribution.

b In Ventura et al. were compared attenders with notattenders in FIT screening.

Considering the Finnish model version, CRC mortality reductions due to screening were simulated in line, respectively, with the results of the NORCCAP trial (simulated RR = 0.71 and observed RR = 0.73, 95% CI: 0.56–0.94) and the Finnish population-based study (simulated RR = 0.92 and observed RR = 1.04, 95% CI: 0.84–1.28; Table 3). CRC cumulative mortality was underestimated by the Finnish model replicating the screening arm of NORCCAP trial (at 4 years of follow-up; Figure 2), whereas CRC cumulative incidence was underestimated and overestimated, respectively, in the controls of the gFOBT population-based study (between 4 and 6 years of follow-up) and in the screening arm of the NORCCAP trial (after 10 years; Supplementary Methods).

All three models were developed assuming the same parameters of the Dutch model version for the adenoma progression and the preclinical duration of CRC (the recalibration of those model structural parameters was not performed).

## Discussion

In this study, we developed three European regional MISCAN-Colon model versions varying only a minimum set of model parameters (adenoma onset and CRC stage distribution at diagnosis). Those versions of the model accurately estimated country-specific CRC stage distribution, incidence, mortality rates (in the prescreening period; all model versions), and cancer-specific mortality reduction due to screening (only Italian and Finnish model versions). Using the same adenoma progression times and preclinical duration of CRC across quite diverse countries and screening settings, our findings may suggest that the natural history of CRC does not vary remarkably across the European countries.

Model validation is an important process in the model development, providing measurable insights on the capacity of the models to correctly estimate and predict the disease-specific outcomes and the potential benefits of screening. Five levels of model validity have been proposed by Eddy et al.: face, internal, cross, external, and predictive validity.^30^ In this study, we assessed the validity of our model versions using two of those levels: the internal (for Italy, Finland, and Slovenia) and the external validity (for Italy and Finland). We found consistent results when we validated our model versions. However, some specific outcomes were not predicted within the 95% confidence interval of the corresponding observed outcome. CRC incidence rates without screening were overestimated by the Italian and Slovenian model version among individuals aged 85 years or older. A likely explanation for this discrepancy is the potential underreporting of CRC at older ages due to frailty and comorbidities of this elderly population.^31^ The model also overestimated CRC incidence in the control group of the Finnish gFOBT screening study (Supplementary Methods). One explanation may be the potential contamination of screening in the control group, especially in the early years of the RCT. This hypothesis is supported by the study of Maklin et al. showing that the colonoscopy use in the control group was nearly two thirds of that in the screening group between 2004 and 2010 and nearly 83% in the first 2 years.^32^ Thus, it may reasonable to expect that in the control group some CRCs may be diagnosed earlier with better survival as a result.

In our external validations, CRC cumulative incidence rates were simulated inconsistent with those observed in the last follow-up years of two studies (FIT Florentine study and NORCCAP trial). As data on CRC risk among nonparticipants in screening was limited, those discrepancies may be a direct consequence of the assumptions made to incorporate that lacking information in our model versions. Finally, we also found that the number of CRC deaths predicted by the Italian model version was higher than the actual number observed in the SCORE trial, while CRC mortality outcomes were consistently predicted for the FIT Florence study. It might seem conflicting, but it may be explained considering the input data used in the model. As MISCAN-Colon does not incorporate changes in CRC relative survival over time (only age-, stage-, and localization-specific differences),^9^ we informed our model using CRC relative survival data observed in the years close to the study’s randomization period. However, in Italy, the 5-year CRC relative survival sharply increased (from 53.9% to 59%/61% [colon/rectum cancer]) during 1994 to 2007,^25,27^ resulting in an overestimation in replicating CRC deaths of the SCORE trial. In contrast, in Florence, the 5-year CRC relative survival was more stable with small improvement during 1995 to 2004,^33^ allowing, therefore, accurate CRC mortality model replications.

Model consistency was evaluated considering the 95% confidence intervals. This decision could be disputed, arguing that models and corresponding predictions could have benefited using more narrow confidence intervals (i.e., 50% CI) in the validation process. However, one should be careful with such considerations. CI represents the level of the plausibility of an estimation: from an inference point of view, a 50% CI reflects an interval for which we are 50% confident that the real study value falls within its limits. Using a narrower CI threshold in our model validation may lead us to select more specific models (good fit with the select study) but with lower confidence in their inference (50% confident to be close to the real “population” value). Hence, we decided to use the established 95% CIs and jointly validate our models against several validation targets (simultaneously), providing more robustness and confidence in our model structure.

Validation assessments, as performed in our study, are important in making a microsimulation model clinically useful.^6^ MISCAN-Colon model is now validated against 6 of 9 RCTs included in the Cochrane Library on the benefits of CRC screening: 3 of 4 gFOBT trials,^34–36^ and, including the findings of this study, 3 of 5 FS trials.^15,21,29^ Model validation in the remaining 2 FS trials may not be performed due to, respectively, the frequent occurrence of opportunistic screening and the small number of participants.^37,38^ Moreover, MISCAN-Colon model is now also validated using population-based results on the effectiveness of FIT and gFOBT screening, with successfully fitting outcomes.^26,28^

Still, some limitations are noteworthy. First, when validating our models, we did not assume different screening tests sensitivity according to the location of adenomas. Some studies indicated that sensitivity of stool tests might vary between right-sided and left-sided premalignant lesions, but there is not a full medical consensus on this hypothesis.^37,39,40^ Second, our models were not stratified by gender. Third, MISCAN-Colon does not currently simulate adenoma histology (villous histology or advanced atypia). Thus, after a follow-up colonoscopy investigation, we categorized low- and high-risk adenoma individuals for post-colonoscopy surveillance using the number and size of the found adenomas. Fourth, we informed our models with data collected in absence of screening. Although that data could be considered outdated, it guaranteed a reliable model calibration without needing detailed information on the screening program (i.e., implementation, invitation, adherence, management, protocols, and regional heterogeneity). Finally, the Slovenian MISCAN-Colon model version was only internally validated, limiting therefore the extrapolation of our modelling results to the Eastern European region. However, in our study we showed that MISCAN-Colon was internally validated in all European regions. In addition, the Slovenian model version was calibrated using the same process of the other model versions included and validated in this study. Hence, given the currently available evidence, we think that MISCAN-Colon can also be a useful tool for evaluating CRC screening also in the Eastern European region.

Notwithstanding these limitations, our results have important clinical implications. We derived our models with the same assumptions on adenoma progression and preclinical duration of CRC (important parameters for simulating the adenoma-carcinoma sequence) as calibrated and validated for the Dutch version of the MISCAN-Colon model. Rutter et al. have shown that this assumption may have a substantial impact on the external validity of the MISCAN-Colon model. However, our study suggested that those parameters can be reliable across different model versions. This might indicate that the natural history of CRC may not vary substantially across Europe: when differences in CRC incidence rates are present between countries,^14^ those may be assumed as related to country-specific differences in onset of adenomas (i.e., different prevalence of well-known CRC-associated lifestyle factors) rather adenoma-carcinoma progression. The findings of our study might support some considerations in scientific literature. Since 1988, increasing trends in CRC incidence were observed in 26 European countries due to societal changes in lifestyle factors, such as diet, obesity, and low physical activity.^41^ Biologically, it might be possible that CRC-associated risk factors are influencing merely the onset of adenomas, whereas the effects of the risk factors on the adenoma progression rates are unlikely. This might be seen investigating the associations between lifestyle factors and, respectively, incidence of CRCs and occurrence of adenomas. Only small differences in the relative risks (effect size of the associations) were observed between association to CRC and to adenoma.^42–45^ Our modelling results have also important future implications for MISCAN-Colon because further standardizations, such as an online model version, are therefore possible. For instance, country-specific differences may be included in the MISCAN-Colon model merely adjusting the adenoma onset parameters (with multiplicative factors, as done in the external validations included in this study). EU-TOPIA is currently using the findings of this study to structure an online tool that allows users to upload and use their country-specific data (demographic, epidemiological, and CRC screening information) for simulating and monitoring future benefits of CRC screening (the EU-TOPIA evaluation tool; http://miscan.eu-topia.org). European stakeholders will be able to quantify short- and long-term impacts of CRC screening in their countries. Furthermore, the EU-TOPIA evaluation tool allows users to provide the last available epidemiological and screening data (i.e., CRC survival, invitation coverage, or target population) for providing reliable future predictions (period 2020–2050). From a policy perspective, this tool can help quantify the effects of current limitations in the CRC screening program, evaluate the impact of removing those limitations, and define appropriate budgets and roadmaps for reducing the burden of CRC. However, our modelling findings (and the EU-TOPIA evaluation tool) are specific to Europe. Although MISCAN-Colon seems to be reliable across different populations and screening settings, further assessments are needed before extrapolating the modelling results of our model structure to populations of other continents such as Asia, America, and Africa. Nevertheless, the methodology developed in this study (the calibration and validation process) could be extended to other parts of the world. We have successfully developed MISCAN-Colon model versions for the United States, Canada, and Australia (a model version for China is currently under development). Taking these models as a base, similar online evaluation tools could be developed, for example, for different states in the United States, or countries in Asia.

In conclusion, our findings corroborate the MISCAN-Colon reliability in supporting decision making on CRC screening, especially among European countries. Furthermore, our study provides the regional model versions and the modelling results that can be used to structure an additional online tool able to quantify, monitor, or improve CRC screening in Europe.

## Supplemental Material

sj-doc-1-mpp-10.1177_2381468320984974 – Supplemental material for Development and Validation of Three Regional Microsimulation Models for Predicting Colorectal Cancer Screening Benefits in Europe Click here for additional data file. Supplemental material, sj-doc-1-mpp-10.1177_2381468320984974 for Development and Validation of Three Regional Microsimulation Models for Predicting Colorectal Cancer Screening Benefits in Europe by Andrea Gini, Maaike Buskermolen, Carlo Senore, Ahti Anttila, Dominika Novak Mlakar, Piret Veerus, Marcell Csanádi, Erik E. L. Jansen, Nadine Zielonke, Sirpa Heinävaara, György Széles, Nereo Segnan, Harry J. de Koning and Iris Lansdorp-Vogelaar in MDM Policy & Practice
