# Supplementary material for: Development and Validation of Three Regional Microsimulation Models for Predicting Colorectal Cancer Screening Benefits in Europe
Source: MDM Policy Pract. 2021 Jan 29;6(1):2381468320984974. doi: 10.1177/2381468320984974 (PMC7863172; doi:10.1177/2381468320984974)
Supplement: sj-doc-1-mpp-10.1177_2381468320984974 – Supplemental material for Development and Validation of Three Regional Microsimulation Models for Predicting Colorectal Cancer Screening Benefits in Europe [file sj-doc-1-mpp-10.1177_2381468320984974.doc]

**Supplementary Methods**

CONTENTS

[MISCAN-Colon model overview 2](#__RefHeading___Toc25672927)

[Dutch MISCAN-Colon model version 9](#__RefHeading___Toc25672928)

[Model calibration and validation 10](#__RefHeading___Toc25672929)

[CRC relative survival model adjustment 1](#__RefHeading___Toc25672930)4

[Selection of data for external validation 1](#__RefHeading___Toc25672931)4

[Calibration of the Finnish gFOBT sensitivity and specificity 1](#__RefHeading___Toc25672932)8

[Cancer registry data used for calibrating the Italian model version 1](#__RefHeading___Toc25672933)9

[Additional results for the Italian and Finnish model versions (cumulative colorectal cancer incidence rates – external validation)](#__RefHeading___Toc25672934) 20

[References 2](#__RefHeading___Toc25672935)1

# MISCAN-Colon model overview

MISCAN-Colon is a stochastic, semi-Markov microsimulation model. In a microsimulation model, individuals are simulated one at a time instead of as proportions of a cohort. The advantage of this is that new events can be dependent on past events of that individual, giving the model a ‘memory’. The model is stochastic, which means that sequences of events are simulated by drawing from distributions of probabilities and durations instead of using fixed values. Therefore, the outcomes of the model are subject to random variation. MISCAN uses the Monte Carlo method to simulate all events in the program. Possible events are birth and death of a person, adenoma incidence and transitions from one state of disease to another. MISCAN–Colon consists of three parts (Supplementary Figure 1): demography; natural history; and screening part. These parts are not physically separated in the program, but it is useful to consider them separately.


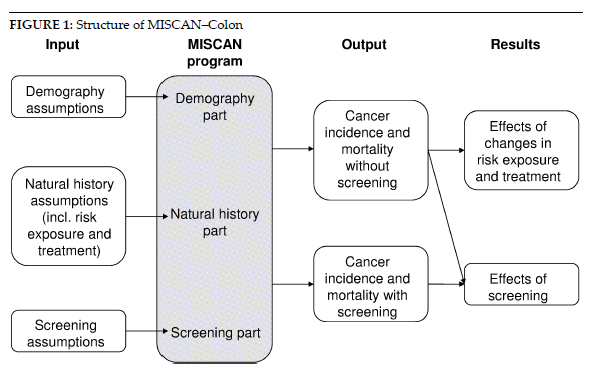


**Supplementary Figure 1**. Structure of MISCAN-Colon

Demography part

MISCAN–Colon first generates a series of individual life histories in the demography part to form a population according to the Demography Parameters. Each person in the population consists of a date of birth and a date of death from other causes than colorectal cancer. These dates are drawn from birth and life tables that are representative for the population under consideration. The maximum age that a person can reach in the model is set to 100 years.

Natural history part

The natural history part of MISCAN–Colon simulates colorectal cancer histories (natural histories) for each individual life history separately. We based our natural history model on the adenoma–carcinoma sequence of Morson and Vogelstein.(1, 2) This means that adenomas are generated according to a personal risk index and an age specific incidence rate. For each person, a risk index is generated at the beginning of the simulation. Based on the risk index and the age specific incidence rate, the ages at which adenomas develop are generated. This results in no adenomas for most persons and one or more adenomas for others. Some of these adenomas develop into colorectal cancer. The development from adenoma into cancer covers different stages and depends on the type of adenoma (non–progressive/progressive), the transition probabilities and the duration distribution. During each invasive preclinical stage, a cancer may be clinically detected because of symptoms before it progresses to a higher stage. The average duration of the preclinical cancer stages and average duration between the adenoma onset and the progression into preclinical cancer (adenoma dwell time) were calibrated using data obtained from randomized, controlled trials (RCTs) evaluating screening(3-7) and recently validated using the NORCCAP trial results.(8) In addition, the model assumes: an equal overall dwell time for adenomas to develop into cancer from medium (30% of all CRCs) and from large size adenomas (70% of all CRCs); an exponential distribution for durations in the adenoma and preclinical cancer states; a perfect correlation between durations within adenoma and preclinical cancer states (quicker growing from small adenoma to medium/large adenoma, faster progression into preclinical CRC); and no correlation between durations within adenoma states and duration in the preclinical cancer states.

Adenomas and cancers are modelled to be continuously distributed over the colorectum. The possible transitions between the different states are represented in Supplementary Figures 2. Once an adenoma has developed into clinical colorectal cancer, the corresponding survival time is dependent on age-, stage-, and localization-specific survival probabilities based on Cancer Registry data.


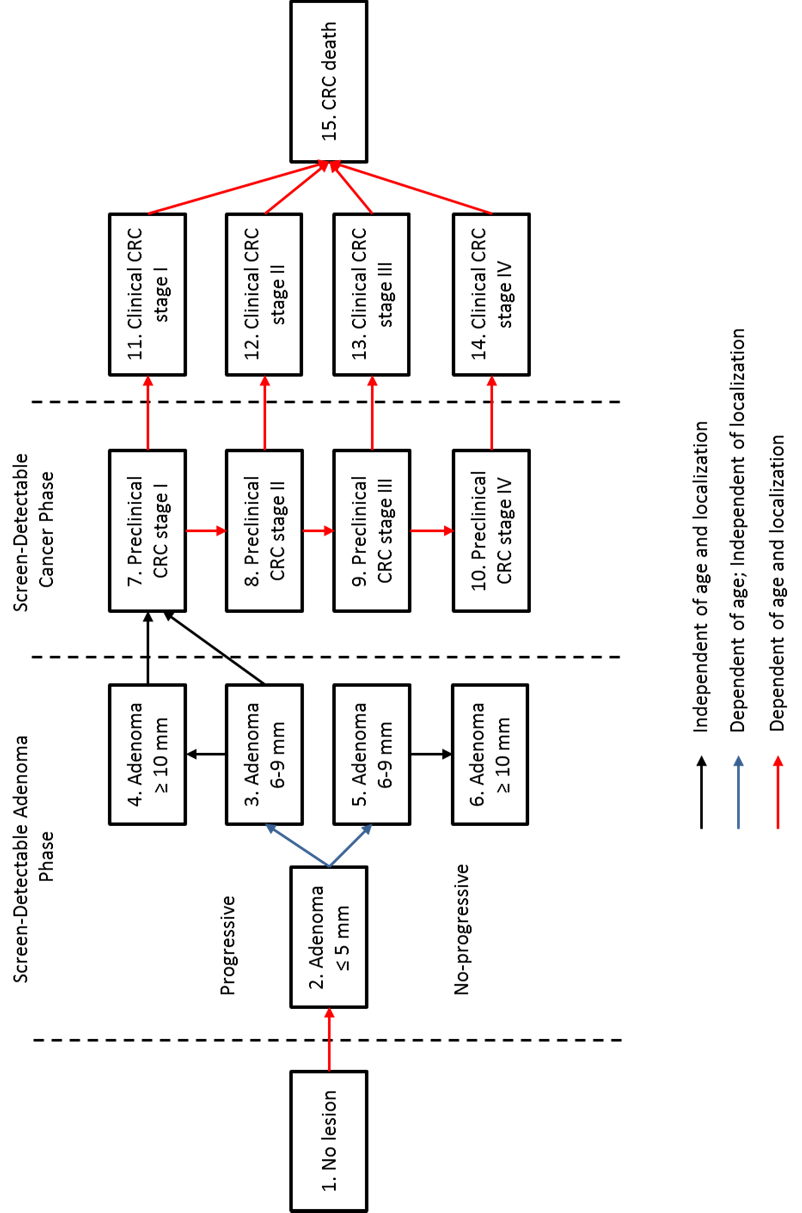


**Supplementary Figure 2**. Model structure with adenoma-carcinoma sequence for progressive adenomas and non-progressive adenoma sequence

The life history of each person is altered according to the colorectal cancer histories (natural history) that is simulated for that person. This means that the state a person is in is the same as the state of the most advanced adenoma or carcinoma he has. If he dies from colorectal cancer before he dies from other causes, his death age is adjusted accordingly. This procedure is explained in Supplementary Figure 3. In this example the life history of a person is shown who develops two adenomas. One of these adenomas develops into a cancer and causes death before the age of death from other causes. The combination of life history without colorectal cancer and the development of adenomas is shown in the bottom line: combined life history for colorectal cancer.

**Supplementary Figure 3**. Modelling natural history into life history

Screening part

In the third part of the program, screening for colorectal cancer is simulated. After the life history of a person is adjusted for colorectal cancer, the history will now be adjusted for the effects of screening. The screening part is simultaneously run with the natural history part, making detection of adenomas and carcinomas in different states possible. Persons can be invited to participate in screening at specified ages as defined in the screening policy. Depending on the test used and the presence of adenomas and/or carcinomas at the moment of the screening test, there is a probability of a positive test result. Screening may detect all non-invasive adenomas and invasive carcinomas, but individual lesions may also be missed. A positive screening test will result either in removal of an adenoma and preventing CRC or early detection of a preclinical carcinoma, possibly in an earlier stage than when it would have been clinically detected, resulting in a favorable stage shift and potentially improved prognosis. The model also incorporates colonoscopy-related complications,(9) over-diagnosis, and overtreatment.

An example of the effect of screening, screening benefit, or over-diagnosis on the life history of an individual is explained in Supplementary Figure 4.


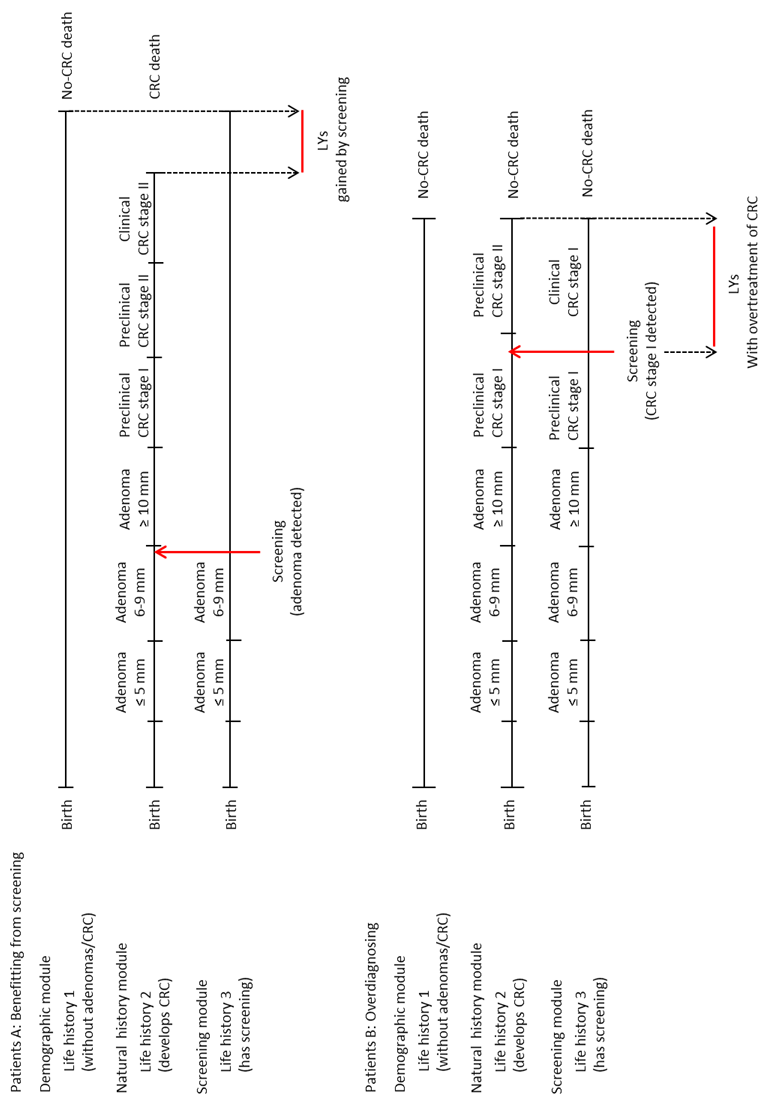


**Supplementary Figure 4**. Modelling screening into life history

In the case of patient A in Figure 4, the natural history part generates an adenoma. This adenoma progress into preclinical cancer and is diagnosed at stage II due to symptoms. This patient dies from CRC before its pre-generated date of death of other causes. The red arrow shows the moment that a screening examination is introduced. In this case the adenoma will be detected, removed, and CRC death is averted. The positive effect of the screening is represented by the red horizontal line, indicating the increase in life years that is gained with the introduction of screening. However, screening might also result in overdiagnosis and overtreatment of CRC (no LYs gained, but only additional LYs with CRC care) as reported in the patient B example. He develops an adenoma that would never have been diagnosed in a no screening scenario. However, during the screening examination, CRC is detected in stage I, resulting in unnecessary treatment.

Besides, an improvement in survival because of stage-shift (i.e. a cancer diagnosed in an earlier stage with screening than without screening), we also assume the possibility for improved survival because of a shift within stage. This is because, as seen in RCTs on guaiac fecal occult blood testing, stage-specific survival in screen-detected CRC, even after the lead-time bias correction, results more favorable compared to clinically detected CRC.(10) In the model, we assign those screen-detected cancer cases that would have been clinically detected in the same stage a survival corresponding to a cancer that is one stage less progressive. Hence, a cancer screen-detected in stage II that would also have been clinically diagnosed in stage II is assigned the survival of a clinically diagnosed stage I cancer. The only exception is made for the screen-detected stage IV cancer cases: we assigned a survival of clinically diagnosed stage IV CRC in those cases.

Model parameters overview

*Demography part*

1. Number of birth cohorts

2. Proportion of the population in each birth cohort

3. For each birth cohort parameters of its birth table

4. For each birth cohort the parameters of its life table

*Natural history part*

1. Adenoma-carcinoma sequence states

2. Age specific adenoma incidence rate by birth cohort

3. Parameters for the distribution of the individual risk index

4. Distribution of adenomas over the colorectal sites

5. Probability for adenomas to be progressive

6. Parameters for the transition probability of non-progressive adenomas for each state

7. Parameters for the duration distribution of non-progressive adenomas for each state

8. Parameters for the transition probability of progressive lesions for each state

9. Parameters for the duration distribution of progressive lesions for each state

10. Correlation between duration in subsequent states

11. Parameters for survival after clinical diagnosis by age at diagnosis, year of diagnosis, stage of disease and localization of the cancer.

*Screening part*

1. Parameters for the dissemination of screening

2. Reach, sensitivity, specificity of different screening tests

3. Dependency of test outcomes on previous test outcomes of the same individual

4. Parameters for survival after screen detected diagnosis

5. Surveillance after screen-detected adenomas

*Parameter nature and distinction*

The parameters reported in the previous section can be divided into three categories (Supplementary Table 1):

• Parameters that are directly estimated from available data

• Parameters for which no data (or limited data) are available

• Parameters that will be varied to fit reference data

**Supplementary Table 1**. Parameters division

| **Parameters that are directly estimated from available data** | **Parameters for which no data (or only limited data are available)** | **Parameters that will be varied to fit reference data (calibrated)*** |
| --- | --- | --- |
| Demography | Transition probabilities from preclinical non-invasive states | Probability for an adenoma to be progressive |
| Distribution of lesions over large bowel | Correlation between durations in subsequent states | Individual risk index |
| Survival after clinical diagnosis | Survival after screen detected diagnosis | Incidence rate of adenomas |
| Distribution of cancers over invasive stages | - | Duration distribution in preclinical states |
| Sensitivity, specificity and reach of screening tests | - | Transition probabilities from preclinical invasive states to clinical states |
| Participation in screening, diagnostic follow-up and surveillance | - | Dependency of test outcomes |
| Relative risk associated with risk and protective factors | - | - |

* for the model versions included in this study, we reported the most relevant calibrated parameters in Supplementary Table 2

# Dutch MISCAN-Colon model version

The Dutch version of the MISCAN-Colon model was first calibrated to age- and stage-specific (UICC TNM stage classification) CRC incidence rates observed in the Netherlands in 1999-2003 (Supplementary Figure 5).(11) Survival rates were based on data from the South of the Netherlands,(11) since nationwide data were not available. The model parameters not directly observable in epidemiological studies, such as adenoma dwell time and the preclinical duration of CRC, were calibrated replicating outcomes of CRC screening RTCs(3-7) and, subsequently, validated to the results of the NORCCAP trial.(8) The Dutch MISCAN-Colon model version has been used to inform the Dutch FIT CRC screening programme(12) and to assess cost-effectiveness of CRC screening.(13-18)

**Supplementary Figure 5**. Model predicted and observed colorectal cancer (CRC) incidence and mortality rates in The Netherlands, 1999-2003.

# Model calibration and validation

As described in Supplementary Table 1, model parameters could not be derived entirely from observed data (only first column of Supplementary Table 1). Developing a country-specific model is not a linear and direct process. Some parameters need to be calibrated based on observed data (third column of Supplementary Table 1), whereas some others need to be assumed and tested comparing model predictions and observed data in screening effectiveness RCTs (second column of Supplementary Table 1). In the past, the MISCAN-Colon model has been calibrated and adapted to reproduce and quantify CRC and CRC screening outcomes in several countries (The Netherlands, US, Canada, and Australia). In Europe, it was used to monitor the Dutch CRC screening programme, predicting its future benefits.(12)

In our study to develop a reliable web-based tool of the MISCAN-Colon model for all of Europe, we developed and validated three new country-specific (Italy, Slovenia, and Finland) model versions. The models were internally and externally validated. Four published studies were selected to perform an external validation of the model: the “once-only” sigmoidoscopy screening or Screening for COlon REctum [SCORE] trial;(19) the cohort study assessing FIT screening in Florence;(20) The Norwegian Colorectal Cancer Prevention [NORCCAP] Trial;(21) and the Finnish gFOBT screening population-based study.(22) For each model we assumed the same natural history parameters (i.e. parameters describing the progression from adenoma to preclinical cancer) of the Dutch model,(7, 8) and only calibrate adenoma onset (CRC incidence) and CRC stage distribution at diagnosis. Only if models developed this way would not validate against observed data, we relaxed this last assumption and re-calibrated the natural history parameters. For more details on the calibration process and on the study selection (external validation) please see the following sections.

The calibration process

To calibrate each new country-specific MISCAN-Colon model version, we used a specific calibration process composed of four steps (Supplementary Figure 6):

1. We started using a previously calibrated model (Dutch MISCAN-Colon model version; for simplicity model A in Supplementary Figure 6) and adjusted specific demographics and CRC epidemiological assumptions, such as population size, age-distributed, all-cause mortality, CRC relative survival by stage and cancer localization (parameters in the first column of Supplementary Table 1). Adjustments for CRC relative survival were performed in comparison with 5-years CRC relative survival observed for The Netherlands in 2000-2007 (for more information, please see the following specific section).
2. We calibrated the parameters behind adenoma onset and probability of CRC diagnosis in each stage. Calibration was performed using CRC incidence rates and CRC stage distribution (reported in pre-screening period, also called calibration targets; parameters in the third column of Supplementary Table 1). The Nelder-Mead algorithm was used for calibration in this step.(23)
3. We used the calibrated model in step 2 to replicate the specific CRC incidence and mortality outcomes observed in the selected studies on CRC screening effectiveness (data not used in the calibration process, external validation). Briefly, we adjusted the models to reflect specific demographics, CRC epidemiological, and screening protocol assumptions, such as population size, age-distribution, all-cause mortality, CRC relative survival by stage, screening starting age, stopping age, screening modality, interval, participation, and surveillance protocols in accordance with the selected published studies. Then, we compared the model predicted outcomes with the study observed outcomes. If the model predicted outcomes are within the 95% confidence interval of the corresponding study’s observed outcomes, we proceeded with step 4. Otherwise, we turned back to step 2 re-calibrating the model parameters included in the second and third column of Supplementary Table 1 (the natural history parameters).
4. We obtained a calibrated and validated country-specific MISCAN-Colon model version (called model B in Supplementary Figure 6, for simplicity). The parameters of the calibrated and validated models for The Netherlands, Italy, Slovenia, and Finland are reported in Supplementary Table 2.


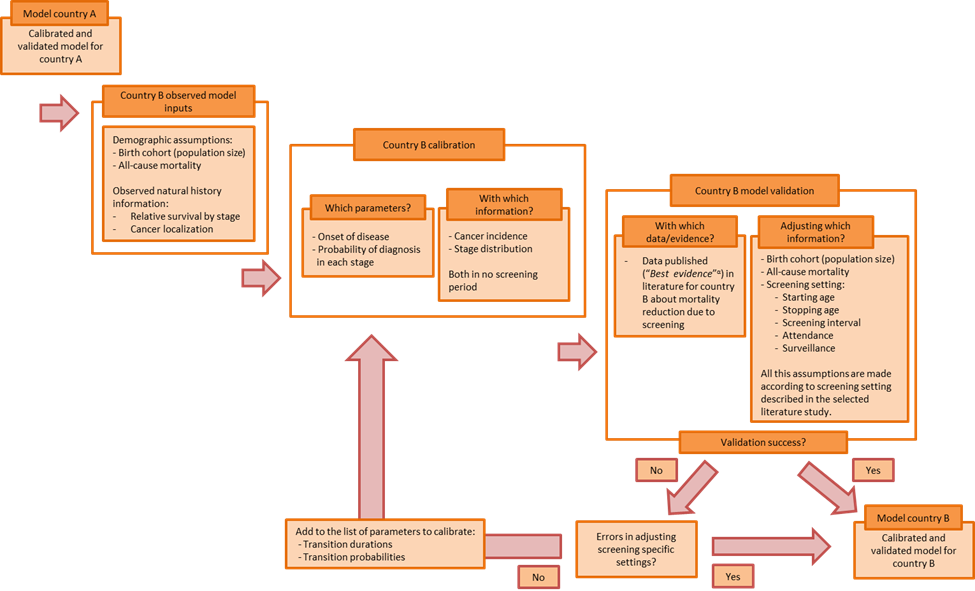


**Supplementary Figure 6**. Calibration and validation process for developing country-specific MISCAN-Colon model version.

**Supplementary Table 2.** Main natural history assumptions in the MISCAN-Colon model

| Model parameters | Model version | | | | Source |
| --- | --- | --- | --- | --- | --- |
|  | The  Netherlands | Italy | Finland | Slovenia |  |
| Distribution of risk for adenomas over the general population* | Gamma distributed, mean 1, variance 2.67 | | | | Fit to multiplicity distribution  of adenomas in autopsy studies(4, 24) |
|  |  |  |  |  |  |
| Adenoma incidence per year |  |  |  |  | Calibrated using CRC incidence data in the pre-screening period(25-28) |
| Age: |  |  |  |  |  |
| 0-20 years | 0.2% | 0.1% | 0.3% | 0.1% |  |
| 20-25 years | 0.3% | 0.2% | 0.3% | 0.2% |  |
| 25-30 years | 0.3% | 0.4% | 0.4% | 0.5% |  |
| 30-35 years | 0.5% | 0.8% | 0.5% | 1.1% |  |
| 35-40 years | 1.2% | 2% | 0.8% | 1.6% |  |
| 40-45 years | 2.8% | 2.7% | 1.3% | 2.5% |  |
| 45-50 years | 3.1% | 2.9% | 2% | 3.4% |  |
| 50-55 years | 3.3% | 2.9% | 2.1% | 3.4% |  |
| 55-60 years | 3.3% | 3% | 2.1% | 3.4% |  |
| 60-65 years | 3.3% | 3% | 2.1% | 3.4% |  |
| 65-70 years | 3.3% | 3% | 2.1% | 3.4% |  |
| 70-75 years | 3.3% | 3% | 2.1% | 3.4% |  |
| 75-80 years | 3.7% | 2.4% | 2.4% | <0.1% |  |
| 80-85 years | 0.3% | 0.1% | 1.2% | <0.1% |  |
| 85-100 years | 0.2% | 0.1% | 0.6% | <0.1% |  |
|  |  |  |  |  |  |
| Probability that a new adenoma  is progressive* | Dependent on age at onset:  0–45 years: 22%  45–100 years: linearly  increasing from 22% to 99% | | | | Fit to multiplicity distribution  of adenomas in autopsy studies(4, 24) |
| Regression of adenomas* | No significant regression of  Adenomas | | | | Expert opinion |
| Mean duration of development of  progressive adenomas to preclinical cancer (Considering all simulated individuals with progressive adenomas)* | 140 years**  **Case 1. Medium adenoma 🡪 preclinical CRC:**  Small to medium adenoma = 99 years  Medium adenoma to preclinical CRC = 41 years  **Case 2. Large adenoma 🡪 CRC:**  Small to medium adenoma = 69 years  Medium to large adenoma = 29 years  Large adenoma to preclinical CRC = 42 years | | | | Rutter et al(7) |
| Mean duration of adenoma (among simulated individuals that developed clinical CRC)* | 14.5 years | | | |  |
| Mean duration of preclinical cancer (among simulated individuals that developed clinical CRC)* | 2.5 years | | | |  |
| Mean duration time from adenoma onset to cancer diagnosis (among simulated individuals that developed clinical CRC)* | 17 years | | | |  |
| Percentage of nonprogressive  adenomas that stay 6-9 mm* | 75% | | | |  |
| Percentage of nonprogressive adenoma  that become 10 mm or larger* | 25% | | | |  |
| Percentage of cancers that develops  from 6-9 mm adenoma and from  10þ mm adenoma* | 30% of cancer develops  from 6-9 mm, 70%  from 10 mm or larger | | | | Expert opinion |
| Localization distribution of adenomas  and cancer |  | | | |  |
| Rectum | 26.4% | 25.3% | 34.7% | 35.3% | Cancer registries data(25-27) |
| Rectosigmoid junction | 9.1% | 10.3% | 6.1% | 10% |  |
| Sigmoid colon | 26.4% | 26.3% | 20.7% | 23.4% |  |
| Descending colon | 6% | 7.6% | 2.8% | 3.1% |  |
| Transverse colon (including flexures) | 9% | 9.4% | 9.2% | 11.4% |  |
| Ascending colon | 8.8% | 8.1% | 26.3% | 8% |  |
| Cecum | 14.3% | 13.1% | 0.2% | 9.1% |  |

* these parameters were assumed the same across the four country-specific model versions of the MISCAN-Colon model (please see calibration process and Supplementary Figure 6); ** not all the progressive adenomas are simulated to progress to preclinical cancer.

# CRC relative survival model adjustment

Parameters of CRC relative survival in MISCAN-Colon need to be informed with age-, stage- (UICC TNM stage), localization-, and year-specific (since cancer diagnosis) data. Unfortunately, such detailed information was not available due to different CRC staging (Finland) or lack of detailed information in the pre-screening period (Italy and Slovenia). In that case, we adjusted the model CRC relative survival parameters comparing the 5-years relative survival observed in the specific country (during the pre-screening period) with the observed survival in the Netherlands during 1999-2013 or 2010-2014 (depending of the value of 5-Year Relative survival observed in the country: <61% (Slovenia, Italy), The 1999-2003 Dutch survival was used; ≥61% (for Finland, and Florence – Italy) The 2010-2014 Dutch survival was used).(29-31) The ratio between overall 5-year CRC survival observed in the specific country (in the pre-screening period) and The Netherlands was, subsequently, used – as multiplicative factor – to adjust the MISCAN-Colon age-, stage-, and localization-specific CRC relative survival model parameters (previously estimated in the Dutch MISCAN-Colon model version).

# Selection of data for external validation

We performed a systematic review was to summarize the effects of colorectal cancer screening on cancer-specific mortality in Europe.(32) Briefly, Six databases including Embase, Medline, and Web of Science were searched for relevant studies published before March 2018. Bibliographic searches were conducted to select studies assessing the effect of various screening tests (guaiac fecal occult blood test, gFOBT; flexible sigmoidoscopy, FS; fecal immunochemical test, FIT; and colonoscopy) on CRC mortality in Europe (PROSPERO Protocol: CRD42016042433). A total of 18 studies were included (3,741 citations were retrieved through the initial searches), of which 11 were related to gFOBT, 4 to FS, 2 to FIT, and 1 to colonoscopy; 8 were randomized clinical trials and 10 observational studies; and, an approximately equal number of studies represented Northern, Western, and Southern European regions. No studies were found for Eastern Europe. Among the studies included in that systematic review, we selected some studies to validate our model versions. Our selection was carried out using a decision algorithm for identifying the best evidence data, judging the level of evidence of each study based on a group of factors, such as country, outcome, study setting, and quality (risk of bias).

We judged each factor assuming a specific hierarchic order (first country, then outcome, study setting, and quality) as follow (and in Supplementary Table 3):

1. Country was the strongest factor for this report as we looked for best data to validate models for exemplary countries.
2. Outcome prioritizes cancer specific mortality for all three cancer sites. Alternatively, incidence is a useful outcome to validate MISCAN-Colon models rated as Level II.
3. Study setting reflects the favoritism of data from actual cancer screening programs over other study settings, including randomized controlled trials and observational studies.
4. Study design / Risk of bias is a combination of study design and risk of bias and is based on the results of the prior quality assessment.

The final results of the selection are reported in Supplementary Table 4. No studies from Eastern Europe were reported in the SR.

**Supplementary Table 3**. Grading level for selecting the best evidence study

| Criteria | Level | Type of studies retrieved |
| --- | --- | --- |
| Country | I | Studies conducted in that specific country (national level). |
|  | II | Studies conducted inside that specific country (regional level). |
|  | III | Studies conducted in neighboring countries within the same European region (national level). |
|  | IV | Studies conducted in neighboring countries within the same European region (regional level). |
|  |  |  |
| Outcome | I | Studies that assessed the effect of screening on CRC mortality reduction. |
|  | II | Studies that assessed the effect of screening on CRC incidence reduction. |
|  | III | Studies that assessed the effect of screening on overall mortality reduction. |
|  |  |  |
| Study setting | I | Screening program evaluation study. |
|  | II | Research study. |
|  |  |  |
| Study design | I | Randomized Control Trials with Low Risk. |
| / Risk of bias | II | Randomized Control Trials with Moderate Risk / Observational studies with Low Risk (score of 8 or 9); |
|  | III | Randomized Control Trials with High Risk / Observational studies with Moderate Risk (score 5 to 7). |
|  | IV | Observational studies with High Risk (score from 0 to 4). |

**Note**: CRC, Colorectal cancer.

**Interpretation**: A study conducting a screening program evaluation within an exemplary country and investigating the impact of screening on cancer specific mortality will be considered as highest level of evidence. However, when no study will show all these factors at the same time the selection will be performed giving priority, respectively, to country, outcome, study setting, and combination of study design and risk of bias.

**Supplementary Table 4***.* Results of our selection for best evidence study for screening modality and country-specific model.

| **Model version** | **Country** | **Outcome** | **Study**  **setting** | **Study design/**  **risk of bias** | **Results (95%CI)** | |
| --- | --- | --- | --- | --- | --- | --- |
|  |  |  |  |  | **Invited vs**  **non-invited** | **Participants vs**  **non-participants** |
| **Stool test (gFOBT/FIT)** |  |  |  |  |  |  |
| **Finland:**  Pitkaniemi J, 2015(22) | I | I | I | II  RCT (B) | RR = 1.04  (0.84-1.28) |  |
| **Italy:**  Ventura L, 2014(20) | II | I | I | II  Cohort (8/9) |  | SMR = 0.59  (0.37-0.93) |
| **Endoscopy test (FS/Colonoscopy)** |  |  |  |  |  |  |
| **Finland:**  Holme Ø, 2014(21) | III | I | II | I  RCT (A) | HR = 0.73  (0.56-0.94) |  |
| **Italy:**  Segnan N, 2011(33) | I | I | II | I  RCT (A) | RR = 0.78  (0.56-1.08) |  |

RCT = Randomized Controlled Trial, RR = Relative Risk, HR = Hazard Ratio, SMR = Standardized mortality ratio, CI = Confidence interval

# Calibration of the Finnish gFOBT sensitivity and specificity

Aiming to validate the Finnish MISCAN-Colon model using the result of the gFOBT population-based screening study in Finland, we performed an additional calibration. We calibrated model parameters behind gFOBT sensitivity and specificity in Finland. This calibration was performed using data from the Finnish Cancer Registry and CRC screening programme: CRC and adenoma detection rates (and positive predictive values for CRC and adenoma) were computed among Finnish individuals invited for the first time to gFOBT screening in 2004-2006. Those rates were used to inform our calibration. Observed and model simulated rates are reported in Supplementary Figure 7. Calibrated parameters for gFOBT sensitivities and specificity are reported in Table 2.

**Supplementary Figure 7**. Simulated and observed CRC and adenoma detection rates (along with positive predicted values, PPV) for Finnish individuals invited for gFOBT screening for the first time in 2004-2006.

# Cancer registry data used for calibrating the Italian model version

| **Supplementary Figure 8**. Cancer registry data included. Italy, 1998-2002 |  | **Supplementary Table 5**. Cancer registry included. Italy, 1998-2002 |
| --- | --- | --- |
| 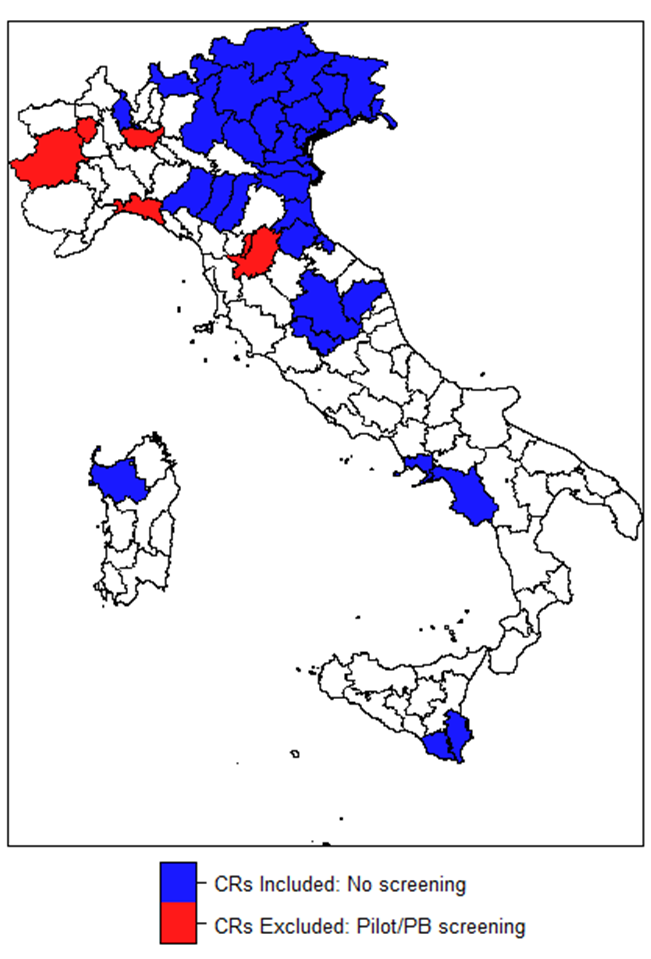  **Notes:** Romagna Region included individuals aged 55-64 invited in SCORE trials (0.6%); Veneto Region provided to IARC data in period 1998-2001 and started FIT screening in May 2002; CRC, colorectal cancer; CRs, Cancer Registries; IARC Cancer Incidence in 5 Continents (CI5-IX).  **Supplementary Table 5** n**otes** (🡪): Marked CRs were excluded in the CRC incidence rates due to early or pilot implementation of CRC screening. |  | | Italian CRs, 1998-2002 | | --- | | Biella Province (1998-2002) | | Brescia Province (1999-2001) | | Ferrara Province (1998-2002) | | Florence and Prato (1998-2002) | | Genoa Province (1998-2000) | | Macerata Province (1998-2000) | | Milan (1999-2002) | | Modena Province (1998-2002) | | Naples (1998-2002) | | North East Cancer Surveillance Network (1998-2002) | | Parma Province (1998-2002) | | Ragusa Province (1998-2002) | | Reggio Emilia Province (1998-2002) | | Romagna Region (1998-2002) | | Salerno Province (1998-2001) | | Sassari Province (1998-2002) | | Syracuse Province (1999-2002) | | Sondrio (1998-2002) | | Turin (1998-2002) | | Umbria Region (1998-2002) | | Varese Province (1998-2000) | | Veneto Region (1998-2001) | |

# Additional results for the Italian and Finnish model versions (cumulative colorectal cancer incidence rates – external validation)

**Supplementary Figure 9**. Simulated and observed cumulative colorectal cancer incidence in SCORE trial, Florentine FIT population-based screening programme, NORCCAP trial, and Finnish gFOBT population-based study. CRC = Colorectal cancer.

# References
